# Forest before the trees in the aquatic world: global and local processing in teleost fishes

**DOI:** 10.7717/peerj.9871

**Published:** 2020-09-24

**Authors:** Maria Santacà, Maria Elena Miletto Petrazzini, Marco Dadda, Christian Agrillo

**Affiliations:** 1 Department of General Psychology, University of Padova, Padova, Italia; 2 Department of Biomedical Sciences, University of Padova, Padova, Italia; 3 Padua Neuroscience Center, University of Padova, Padova, Italia

**Keywords:** Visual hierarchical stimuli, Gestalt, Comparative perception, Navon test, Teleost fish

## Abstract

**Background:**

The study of illusory phenomena is important to understanding the similarities and differences between mammals and birds’ perceptual systems. In recent years, the analysis has been enlarged to include cold-blooded vertebrates, such as fish. However, evidence collected in the literature have drawn a contradictory picture, with some fish species exhibiting a human-like perception of visual illusions and others showing either a reversed perception or no susceptibility to visual illusions. The possibility exists that these mixed results relate to interspecific variability in perceptual grouping mechanisms. Therefore, we studied whether fish of five species exhibit a spontaneous tendency to prioritize a global analysis of the visual scene—also known as global-to-local precedence—instead of focusing on local details.

**Methods:**

Using Navon-like stimuli (i.e., larger recognisable shapes composed of copies of smaller different shapes), we trained redtail splitfin, zebrafish, angelfish, Siamese fighting fish and three spot gourami to discriminate between two figures characterized by congruency between global and local information (a circle made by small circles and a cross made by small crosses). In the test phase, we put global and local cues (e.g., a circle made by small crosses) into contrast to see whether fish spontaneously rely on global or local information.

**Results:**

Like humans, fish seem to have an overall global-to-local precedence, with no significant differences among the species. However, looking at the species-specific level, only four out of five species showed a significant global-to-local precedence, and at different degrees. Because these species are distantly related and occupy a broad spectrum of ecological adaptations, we suggest that the tendency to prioritize a global analysis of visual inputs may be more similar in fish than expected by the mixed results of visual illusion studies.

## Introduction

Visual illusions represent one of the most powerful tools to shed light on our representation of static and dynamic visual stimuli (Gori & Stubbs, 2013; Spillmann, 1994). The main goal of perceptual mechanisms and systems is to detect and interpret sensory inputs for a rapid response to environmental changes. With respect to this concept, visual illusions allow us to analyse and understand the perceptual mechanisms underlying visual perception and, as a consequence, to understand their constraints (Carbon, 2014). Recently, the study of visual illusions has become an important non-invasive approach to investigate and compare the perceptual mechanisms of vertebrates (see Feng et al., 2017 for a review). The evolutionary assumption underlying this type of investigation is that the species that show the same susceptibility to a specific visual illusion, also share similar perceptual mechanisms. The studies conducted until now do not seem to support the universality of the perceptual mechanisms because different species have shown they do not perceive visual illusions or, in a few cases, have had completely different perceptions of a visual illusion. In addition, several species have not been studied yet.

The Delboeuf and the Ebbinghaus illusions are similar, well-known distortion illusions that consist of the misperception of the size of a target circle depending on its surrounding context. Several non-human primate species have been shown to perceive the Delboeuf illusion as humans do, such as chimpanzees (*Pan troglodytes*; Parrish & Beran, 2014) and capuchin monkeys (*Cebus apella*; Parrish, Brosnan & Beran, 2015). A reptile species also showed it perceives this illusion in the same manner as humans do (i.e., bearded dragons, *Pogona vitticeps*; Santacà et al., 2019), whereas a bird species demonstrated it perceives the Ebbinghaus illusion as humans do (i.e., domestic chicks, *Gallus gallus*; Rosa Salva et al., 2013). The human-like perception of these illusions suggests different vertebrate species may share assimilation/contrast effects (Parrish, Brosnan & Beran, 2015). However, other studies reported no sensitivity to these illusory patterns (i.e., baboons (*Papio papio*; Parron & Fagot, 2007) and dogs (*Canis familiaris*; Miletto Petrazzini, Bisazza & Agrillo, 2017; Byosiere et al., 2017)), suggesting that these species do not have the same perceptual mechanisms described in humans. In addition, two different bird species demonstrated they have a reverse perception of the Ebbinghaus illusions: bantams (Nakamura, Watanabe & Fujita, 2014) and pigeons (*Columba livia*; Nakamura, Watanabe & Fujita, 2008). Another well-known visual illusion is the Müller Lyer illusion in which the perceived length of a line is influenced by the surrounding context. Different species, from primates to reptiles, demonstrated they perceive this illusory pattern as humans do: capuchin monkeys (Suganuma et al., 2007), parrots (*Psittacus erithacus*; Pepperberg, Vicinay & Cavanagh, 2008), pigeons (Nakamura et al., 2006) and bearded dragons (Santacà et al., 2020). Regarding the Zöllner illusion, a similar heterogeneous scenario can be found in the literature. The Zöllner illusion consist of perceiving parallel lines as divergent due to the presence of diagonal crossing lines. Rhesus monkeys (*Macaca mulatta*; Agrillo, Parrish & Beran, 2014) and baboons (Benhar & Samuel, 1982) exhibited a human-like perception of the Zöllner illusion whereas bantams (*Gallus gallus domesticus*) and pigeons showed a reverse perception of this illusion (Watanabe, Nakamura & Fujita, 2011; Watanabe, Nakamura & Fujita, 2013).

Concerning the literature on more distantly related species, such as fish, the pattern is still not clear, and it is difficult to establish the extent to which humans and fish share similar perceptual mechanisms (for a review see Agrillo et al., 2020). Studies have shown that fish experience a human-like perception of several illusory phenomena, such as a motion illusion (guppies (*Poecilia reticulata*) and zebrafish (*Danio rerio*): Gori et al., 2014), a brightness illusion (guppies; Agrillo, Miletto Petrazzini & Bisazza, 2016) and the Müller-Lyer illusion (redtail splitfin (*Xenotoca eiseni*); Sovrano, Da Pos & Albertazzi, 2016; Santacà & Agrillo, 2020). However, mixed results have been reported in the presence of other illusory phenomena. It has been found that damselfish (*Chromis chromis*; Fuss & Schluessel, 2017) perceive the Delboeuf illusion in a human-like direction, whereas guppies (Lucon-Xiccato et al., 2019) and bamboo sharks (*Chiloscyllium griseum*; Fuss & Schluessel, 2017) seem to experience a reverse illusion (perceiving as larger the stimulus seen as smaller by human observers). A similar result was found with the Ebbinghaus illusion, in which damselfish (Fuss & Schluessel, 2017) and redtail splitfin (Sovrano, Albertazzi & Rosa Salva, 2015) apparently perceive the illusion like humans while bamboo sharks show little evidence of being susceptible to it (Fuss & Schluessel, 2017).

Before drawing the conclusion that the perceptual mechanisms underlying visual illusions in vertebrates are widely different, we need to take into account a more parsimonious explanation. It is possible that these perceptual mechanisms are more similar than that hypothesized from the results of visual illusion studies. Instead, animals might differ with respect to a more basic perceptual mechanism that affects the overall perception of the visual scene, the global-to-local precedence. Humans prioritize a global perception of the elements; we focus our attention on the ‘forest’ before noticing the ‘trees’ (Navon, 1977). Such a global view is supposed to be a perceptual prerequisite for the perception of several illusory phenomena (e.g., Feng et al., 2017; Kelley & Kelley, 2014). For instance, the perception of the Solitaire illusion is based on Gestalt perception: items forming a single Gestalt are commonly overestimated compared to items forming separate (smaller) clusters (Frith & Frit, 1972). Clearly, this numerosity illusion cannot be experienced by individuals who exhibit a poor global-to-local precedence for reasons that are not directly related to perceptual biases of numerical estimation. Other illusory phenomena are likely to be based on global-to-local precedence: to experience the assimilation/contrast effects by the Delboeuf/Ebbinghaus and the Müller-Lyer illusions, individuals have to focus on the global picture that includes both the target circles or lines and their surrounding contexts (Girgus & Coren, 1982). In fact, if a species has a local-to-global perception of the stimuli, it may only focus its attention on local cues (i.e., only the target circles or lines) resulting in a reduction of the assimilation/contrast effects that disrupt or reduce the illusory effects.

Similar to visual illusion studies, research on global-to-local precedence in non-human species has provided mixed results. Chimpanzees display a rather robust global-to-local precedence (e.g., Fujita & Matsuzawa, 1990; Hopkins, 1997; Hopkins & Washburn, 2002). However, local-to-global precedence was found in capuchin monkeys (De Lillo et al., 2011) even if this species demonstrated it perceives several visual illusions in a human-like manner (for example, the Delboeuf (Parrish, Brosnan & Beran, 2015) and the Müller-Lyer (Suganuma et al., 2007) illusions). Pigeons also demonstrated a local-to-global precedence (Cavoto & Cook, 2001); however, they perceive the Müller-Lyer illusion as humans do but not the Zöllner illusion. Moreover, inter-individual differences in global-to-local precedence have been reported within species too, as the case of dogs (Pitteri et al., 2014).

Although fish represent approximately half of vertebrate species, the study described below is the only one done regarding the perceptual grouping mechanisms in fish. Truppa et al. (2010) investigated whether redtail splitfin exhibit a global-to-local precedence using Navon-like stimuli. Subjects were initially trained with food reward to discriminate between a circle made of small circles and a cross made of small crosses. Both stimuli were characterized by a congruency between global (e.g., a big circle) and local (small circles) information. In the test phase, researchers put global and local information into contrast, presenting a circle made by small crosses and a cross made by small circles. These trials permitted the establishment of the global/local encoding preferences of this species. Results showed that redtail splitfin trained with the circle of small circles as positive stimulus selected the circle made by crosses, whereas fish trained with the cross made by small crosses selected the cross made of circles, thus showing a global-to-local precedence. All studies on visual illusions in redtail splitfin have provided evidence of a human-like perception of different phenomena (reviewed in Agrillo et al., 2020), which strengthens the hypothesis of a link between global-to-local precedence and the susceptibility to visual illusions.

In the present study we extended the investigation of global-to-local precedence in teleost fishes. To achieve this goal, we compared the performance of Siamese fighting fish (*Betta splendens*), zebrafish, angelfish (*Pterophyllum scalare*), three spot gourami (*Trichopodus trichopterus*) and redtail splitfin. This type of investigation is supposed to provide a broad picture of similarities and differences in global-to-local precedence of fish species. These species are distantly related and each belongs to a different subfamily or family; therefore, they have evolved independently for millions of years. Nonetheless, these species are similar in size, life span, feeding and social behaviour. They also underwent a broad spectrum of ecological adaptations and slightly differ in the habitat in which they live (e.g., Roy & Bhat, 2018; Piller et al., 2015). In particular, all species inhabit murky waters with the exception of zebrafish that prefer clear waters (Spence et al., 2006). Zebrafish is also the most distantly related species compared to the other four. Due to their long divergence time and their different ecological needs, this investigation indirectly allows an understanding of the influence of the evolutionary and ecological pressures on the global-to-local precedence. Subjects were initially trained to discriminate between two figures characterized by congruency between global and local information (a circle made by small circles and a cross made by small crosses). As soon as they met the learning criterion, global and local information were contrasted in non-reinforced test trials. For both types of trials, two different densities of local information were arranged to assess whether the variation in the spatial separation of the local figures influences the perceptual grouping mechanisms of fish. Previous experiments demonstrated that the global preference of different species, such as humans and various non-human primate species, was highly affected by the density of the local elements and that, in some cases, a reduction of such density changed the global-to-local precedence into a local-to-global preference (e.g., Fagot & Tomonaga, 1999; Martin, 1979). If fish exhibited a global-to-local precedence they were expected to select the stimuli with the global features of the previous trained stimulus (e.g., a circle made by small crosses, if they were trained to select the circles made by small circles). On the contrary, if they displayed a local-to-global precedence, then they were expected to select the stimulus with the same local information presented in the training phase (e.g., selecting the cross made by small circles if previously reinforced to select the circle made by small circles).

## Materials & Methods

### Subjects and experimental apparatus

**Figure 1:**
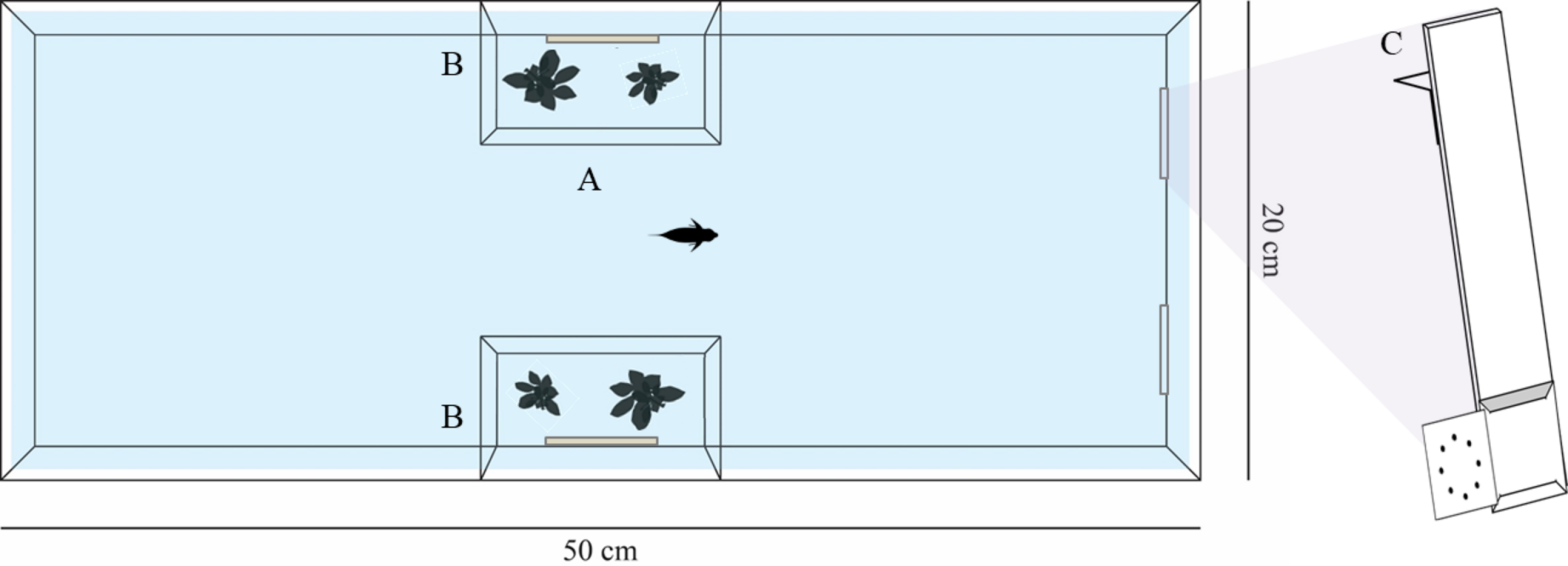
Representation of the experimental apparatus. The experimental tank was composed of a central runaway (A) and two lateral compartments (B) that housed natural plants and a mirror. Each stimulus was presented by means of a transparent support (C).

The experimental design required 12 subjects per species. However, in training experiments some subjects often do not habituate to the experimental approach or do not reach the learning criterion of the training phase. Therefore, such fish have to be substituted with new subjects. As a consequence, we had to test 18 Siamese fighting fish, 13 zebrafish, 14 angelfish, 22 three spot gourami and 22 redtail splitfin. In particular, four Siamese fighting fish, one zebrafish, one angelfish, seven three spot gourami and five redtail splitfin fish were discharged because they did not reach the learning criterion, whereas all other discharged subjects did not start or completed the training phase of the experiment since they did not approach the stimuli or had difficulties doing so. All tested subjects were adult females with the exception of angelfish that were unsexed juvenile individuals. Redtail splitfin came from the stocks regularly maintained in the laboratory at the Department of General Psychology of the University of Padova. Siamese fighting fish, angelfish, three spot gourami and zebrafish were obtained from a local commercial supplier one month prior to the experiment. Each species was housed separately in 110-litre stock aquaria enriched with gravel bottom, natural plants and mechanical filters. Stock aquaria and experimental tanks were maintained at 26 °C and were lit by a 30 W fluorescent lamp (12 h:12 h light/dark photoperiod). Before the experiment, fish were fed twice a day with commercial food flakes (Aqua Tropical, Padovan^^®^^) and live brine shrimp (*Artemia salina)*. The experimental apparatus was successfully adopted in several previous experiments (e.g., Lucon-Xiccato et al., 2019; Santacà & Agrillo, 2020). The experiments were performed in 18 identical glass tanks (50 × 20 × 35 cm). The tank was shaped like an hourglass by two lateral compartments (10 × 6 × 32 cm) in which plants and a mirror were placed (Fig. 1). The plants recreated a natural environment, whereas the mirrors prevented stress due to the social isolation (e.g., Lucon-Xiccato et al., 2019; Santacà & Agrillo, 2020). The four walls of each tank were covered with sheets of green plastic to prevent influence from the external environment on the subjects’ performances. After the experiment, the subjects were released in specific stock tanks and kept for breeding.

### Stimuli

**Figure 2:**
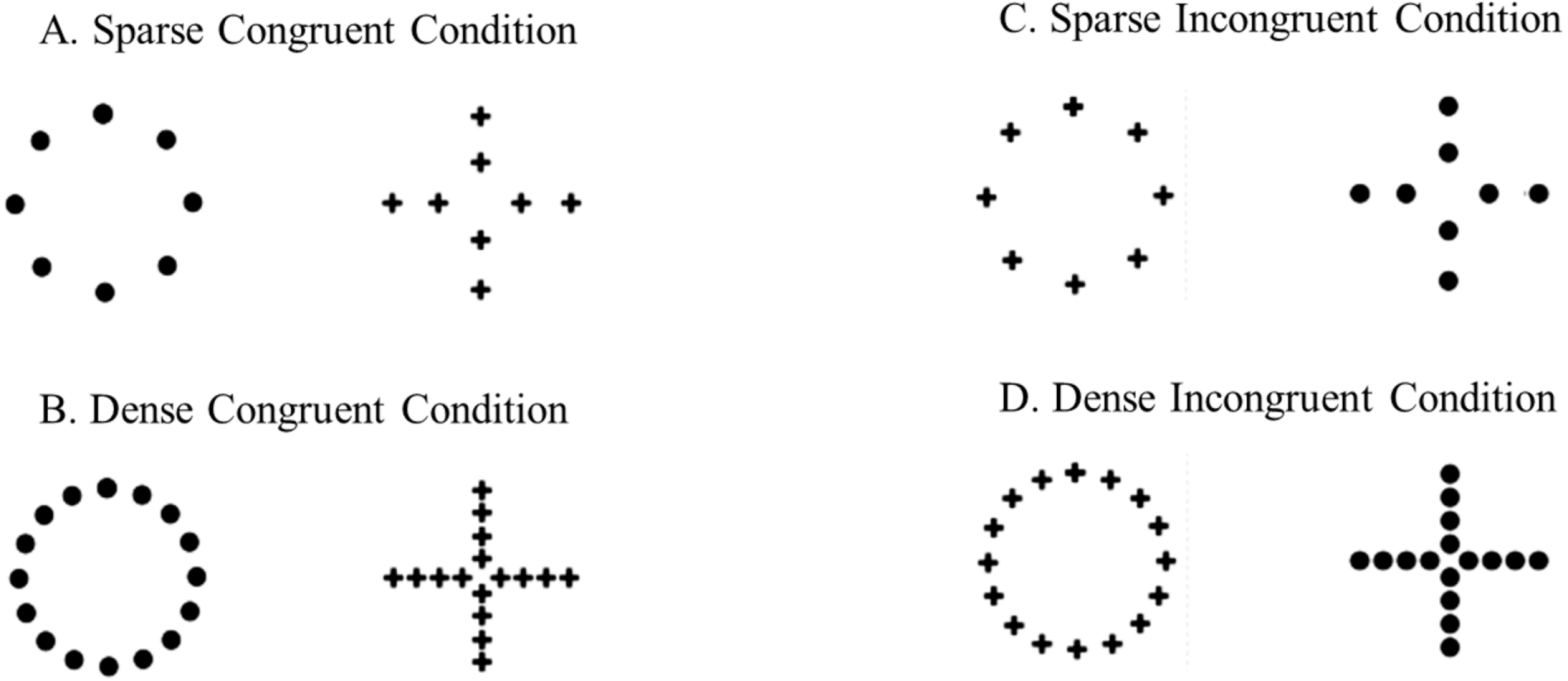
Experimental stimuli. Subjects were trained to discriminate between two stimuli characterized by congruency between global and local information: (A) a circle made by small sparse circles or a cross made by small sparse crosses; (B) a circle made by small dense circles or a cross made by small dense crosses. Reached the learning criterion, they were presented with non-reinforced test trials. Subjects had to choose between two stimuli in which the global and local information were contrasted: (C) a circle made by small sparse crosses or a cross made by small sparse circles; (D) a circle made by small dense crosses or a cross made by small dense circles.

Stimuli consisted of hierarchically structured black figures printed on 3 × 3 cm white cards. During the trials, subjects were presented with two cards that were affixed to the end of a transparent L-shaped support (3.5 × 15 cm; Fig. 1C); two supports were placed on the same short wall of the experimental apparatus. During the training phase, the stimuli presented to the subjects consisted of a big circle (2.3 cm in diameter) made of small circles (0.2 cm in diameter) and a big cross (2.5 cm in length) made of small crosses (0.3 cm in length). These hierarchically structured stimuli composed the congruent conditions because they were characterized by a congruency between the global (e.g., a big circle) and the local (e.g., small circles) information. Two different densities of the elements (small circles or crosses) were arranged: in the ‘Sparse Congruent Condition’, the big figure was composed by 8 small elements, whereas in the ‘Dense Congruent Condition’, the big figure was composed by 16 small elements (Figs. 2A, 2B). In the test phase, two additional types of condition were presented to the subjects, the incongruent conditions. Unlike the congruent conditions, the global and the local information were in contrast: the big circle was made of small crosses and the big cross was made of small circles. As for the congruent conditions, two different densities of the elements were arranged: in the ‘Sparse Incongruent Condition’, the big figure was composed by 8 small elements, whereas in the ‘Dense Incongruent Condition’, the big figure was composed by 16 small elements (Figs. 2C, 2D).

### Procedure

The procedure was adapted from various investigations regarding the perception of visual illusions in guppies (e.g., Lucon-Xiccato et al., 2019; Santacà & Agrillo, 2020) and was composed of two subsequent phases, a training phase and a test phase. Two days before the beginning of the experiment, the subjects were moved in the experimental apparatuses to familiarize them with the new tank.

#### Training phase

The training phase lasted up to 5 days. Every day, each subject performed two sessions of 6 trials each. A 90-minute interval separated the two sessions whereas a 15-minute interval separated two consecutive trials. Half of the tested subjects of each species were trained to select the cross in the congruent condition (6 out of 12): 3 out of 6 were trained using the Sparse Congruent Condition whereas the other 3 were trained using the Dense Congruent Condition. The other 6 out of 12 subjects of each species were trained to select the circle in the congruent condition: 3 out of 6 were trained using the Sparse Congruent Condition whereas the other 3 were trained using the Dense Congruent Condition. If a subject approached the correct stimulus, a small quantity of live shrimps (*Artemia salina*) was provided by means of a Pasteur pipette as food reward while the incorrect stimulus was removed from the water. In the case of an incorrect choice, no food reward was provided and both stimuli were removed from the tank. Only after a fish made a correct choice, the experimenter approached the tank to provide the food reward which avoided the experimenter’s behaviour influencing the subject. A choice was defined as the first stimulus a subject approached within less than one body length. Two undergraduate students unaware of the study hypothesis conducted each trial and collected the data for each subject; in particular, when one student conducted a trial, the other observed that it was performed correctly and that the subject’s choice was correctly noted. The 12 trials were administered following a pseudorandom order, according to which the position (left/right) of the correct stimulus and the short side of the tank (frontal or posterior) were counterbalanced. In addition, the correct stimulus was not presented in the same position and in the same short side of the tank more than twice in a row. The subjects could pass to the test phase if they achieved a learning criterion, namely 18 out of 24 correct choices in two consecutive days, a significant performance at the binomial test. The subjects that failed to achieve the learning criterion, were discharged and substituted with new subjects.

#### Test phase

The test phase lasted 5 consecutive days during which the subjects participated in 12 trials each day, split in two sessions as done in the training phase. Six out of 12 trials consisted of congruent conditions whereas the other six consisted of incongruent conditions. Congruent trials were normally reinforced as they were during the training phase. Incongruent trials were not rewarded irrespective of the chosen stimulus and both stimuli were simultaneously removed to avoid learning bias. Fish trained with the Dense Congruent Condition were tested with the Dense Incongruent Condition whereas fish trained with the Sparse Congruent Condition were tested with the Sparse Incongruent Condition. In case of a global-to-local precedence, the subjects were expected to choose the stimulus with the global features of the previous trained stimulus: for instance, the big circle made by small crosses, if they were trained to select the big circles made by small circles or the big cross made by small circles, if they were trained to select the big cross made by small crosses. The 12 trials were administered following a pseudorandom order, according to which the position (left or right) of the correct stimulus (or of the stimulus chosen in case of a global-to-local precedence) and the short side of the tank (frontal or posterior) were counterbalanced. However, we checked for side bias (left versus right position) and we did not find it for any subjects of each species (paired *t* tests: all *P* values >  .05). In addition, an incongruent condition was always preceded by a congruent condition during each day; therefore, subjects could not perform two incongruent conditions consecutively.

### Statistical analyses

Statistical analyses were performed using R version 3.5.2 (The R Foundation for Statistical Computing, Vienna, Austria, http://www.r-project.org). Regarding the training phase, we conducted a linear mixed-effects model (LMM, ‘lmer’ function of the ‘lme4’ R package) to compare the number of trials necessary to meet the learning criterion between the five species. Regarding the test phase, we conducted intraspecific and interspecific analyses. Regarding the intraspecific analyses, binomial tests (‘binom.test’ function) were performed to assess whether the performances of each species in both congruent (absolute value of choices for the reinforced control stimulus) and incongruent (absolute value of choices for the stimulus chosen in case of a global-to-local precedence) conditions were different from the chance level (0.50). We used Cohen’s *h* as an effect size statistic that is specific for proportions: a Cohen’s *h* of 0.20 represents a small difference between two proportions, a Cohen’s *h* of 0.50 represents a medium difference, a Cohen’s *h* of 0.80 represents a large difference (Cohen, 1988). We calculated the Cohen’s *h* using the ‘ES.h’ function of the ‘pwr’ R package. Regarding the interspecific analyses, we performed a linear mixed-effects model for binomial response distributions (GLMM, ‘glmer’ function of the ‘lme4’ R package) to investigate whether the five species had different performances in both types of conditions. The same model was used to assess the effects of the test day, the density of the elements (sparse or dense) and the type of reinforced stimulus (cross or circle). The model was fitted with the subject ID as a random effect. Subsequently, we performed all pairwise comparisons with the Tukey honestly significant difference tests (Tukey, 1949). In addition, as intraspecific analyses, we also performed another GLMM to assess inter-individual differences within each species. Lastly, we compared the performance of our experiment with a recent work that investigated the same five species’ susceptibility to the Delboeuf illusion (Santacà, Lucon-Xiccato & Agrillo, 2020) to determine a correlation between the two performances with a Pearson correlation test.

### Ethics statement

We followed all applicable international, national, and/or institutional guidelines for the care and use of animals (Italy, D.L. 4 Marzo 2014, n. 26). The study was in accordance with the ethical standards of the institution where the study was conducted and was approved by the relevant ethics committee (Organismo preposto al benessere animale) of the University of Padova (Protocol n. 32/2019).

## Results

### Training phase

Siamese fighting fish needed 43.71 trials (SD = 12.98 trials), zebrafish needed 36.86 trials (SD = 9.94 trials), angelfish needed 42 trials (SD = 12.23 trials), three spot gourami needed 39.43 trials (SD = 9.91 trials) and redtail splitfin needed 40 trials (SD = 14.77 trials) before meeting the learning criterion. The LMM revealed no significant difference between the number of trials necessary to pass to the test phase between the five species (*F*_4,53_ = 0.599, *P* = .665).

### Test phase

**Figure 3:**
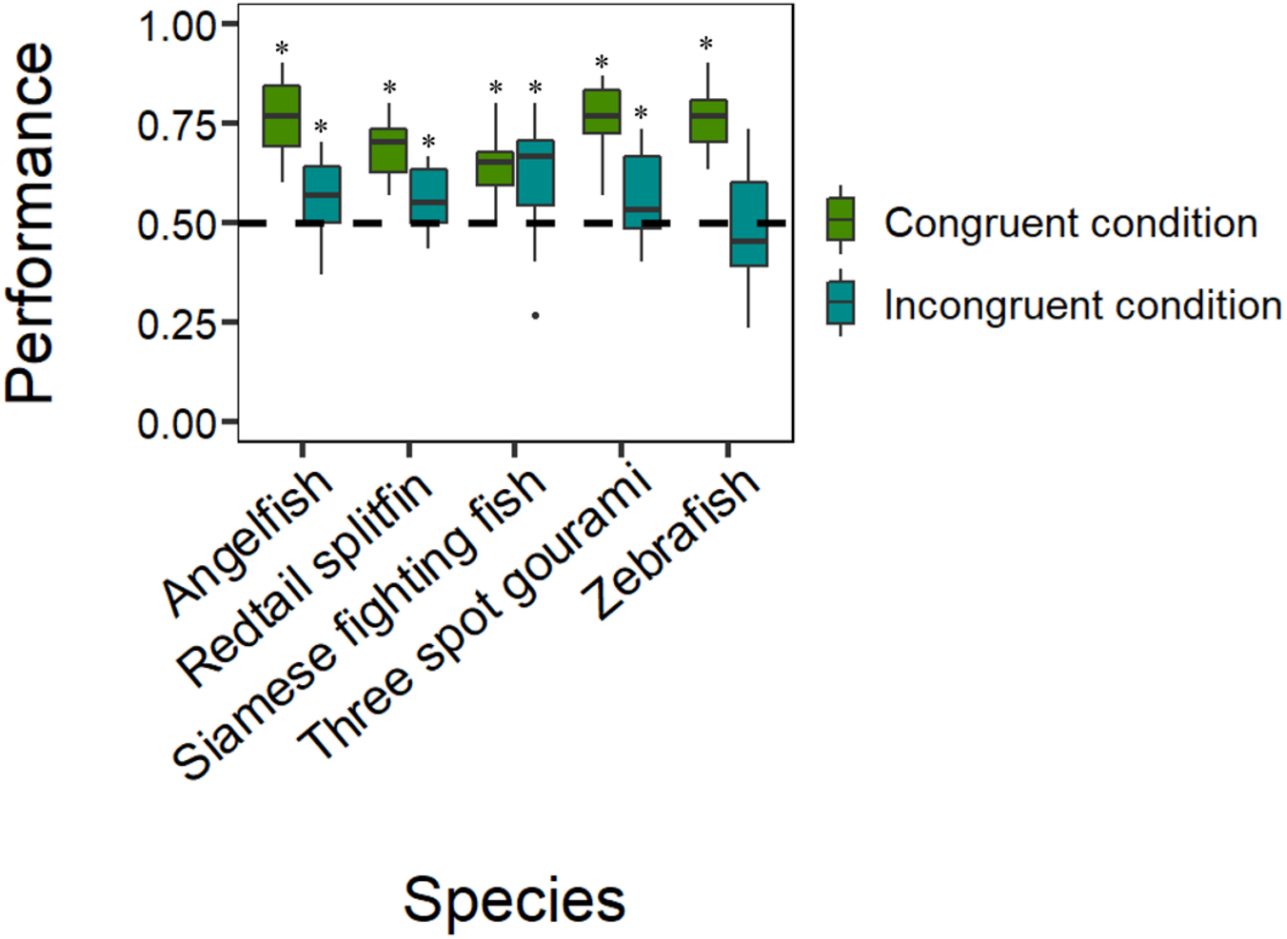
Results. Species-specific boxplots representing median, first quartile, third quartile, ranges, and outliers (data points 1.5 interquartile ranges smaller than the first quartile or greater than the third quartile). The Y-axis refers to the proportion of choices for the reinforced control stimulus in the congruent condition and for the stimulus chosen in case of a global-to-local precedence in the incongruent condition. The asterisk (*) denotes a significant departure from chance level (*P* < .05).

The analyses revealed that all five species selected the reinforced control stimulus in the congruent condition significantly more often than if by chance: Siamese fighting fish (mean: 0.639, 95% CI [0.587–0.689], *P* = 1.518e^−07^, *h* = 0.282; Fig. 3), zebrafish (mean: 0.686, 95% CI [0.635–0.735], *P* = 1.322e^−12^, *h* = 0.381; Fig. 3), angelfish (mean: 0.767, 95% CI [0.719–0.809], *P* < 2.2e^−16^, *h* = 0.563; Fig. 3), three spot gourami (mean: 0.753, 95% CI [0.705–0.796], *P* < 2.2e^−16^, *h* = 0.531; Fig. 3), and redtail splitfin (mean: 0.686, 95% CI [0.635–0.734], *P* = 1.322e^−12^, *h* = 0.381; Fig. 3). The tests on the frequency for the stimulus chosen in case of a global-to-local precedence in the incongruent condition revealed that four species significantly demonstrated they have a global-to-local precedence: Siamese fighting fish (mean: 0.600, 95% CI [0.547–0.651], *P* = .0002, *h* = 0.201; Fig. 3); angelfish (mean: 0.567, 95% CI [0.514–0.619], *P* = .013, *h* = 0.134; Fig. 3), three spot gourami (mean: 0.556, 95% CI [0.503–0.608], *P* = .040, *h* = 0.072; Fig. 3) and redtail splitfin (mean: 0.556, 95% CI [0.503–0.608], *P* = .040, *h* = 0.072; Fig. 3). The remaining species, the zebrafish, did not choose any stimulus more often than chance (mean: 0.464, 95% CI [0.411–0.517], *P* = .562, *h* = 0.072; Fig. 3).

The GLMM showed that the performance did not vary as a function of the species (${\chi }_{4}^{2}=6.924$, *P* = .140), the density of the elements (*χ*^2^_1_ = 0.717, *P* = .397), the type of the reinforced stimulus (${\chi }_{1}^{2}=0.367$, *P =* .545), or the day (*χ*^2^_4_ = 0.201, *P* = .654). The only significant effect was that of the type of condition (*χ*^2^_1_ = 96.615, *P* < 2.2e^−16^), whereas the only significant interaction was that between the species and the type of condition (*χ*^2^_4_ = 24.385, *P* = 6.685e^−05^; all other *P*-values > .069), suggesting that the five species had different performances depending upon the type of condition. In particular, the Tukey post hoc test revealed that the performances of zebrafish, angelfish, three spot gourami, and redtail splitfin in the congruent condition were significantly different from their performances in the incongruent condition (all *P-* values < .02). Instead, the performance of the Siamese fighting fish in the congruent condition was not significantly different from the performance in the incongruent condition (*P* = .990).The Tukey post hoc test revealed that the performance of Siamese fighting fish in the congruent condition was significantly different compared to the performances of angelfish, three spot gourami, and zebrafish (*P* = .006, *P* = .031, and *P* = .008, respectively). Considering the performances in the incongruent condition, the only significant comparison was between the performances of Siamese fighting fish and zebrafish (*P* = .010).

The GLMMs on the intraspecific individual differences revealed a significant difference between the subjects only in zebrafish (${\chi }_{11}^{2}=19.893$, *P* = .047; Siamese fighting fish: ${\chi }_{11}^{2}=13.792$, *P* = .245; angelfish: ${\chi }_{11}^{2}=10.582$, *P* = .479; three spot gourami: ${\chi }_{11}^{2}=14.536$, *P* = .208; redtail splitfin: ${\chi }_{11}^{2}=8.930$, *P* = .628).

The Pearson correlation test revealed that no significant correlation existed between the global-to-local precedence and the perception of the Delboeuf illusion of the five species (*r*_3_ = 0.508, *P* = .382). Siamese fighting fish (mean: 0.422, 95% CI [0.356–0.488]), redtail splitfin (mean: 0.396, 95% CI [0.306, 0.486]), and angelfish (mean: 0.406, 95% CI [0.317–0.495]) demostrated they have a significant reverse perception of the Delboeuf illusion (Santacà, Lucon-Xiccato & Agrillo, 2020). Zebrafish (mean: 0.396, 95% CI [0.277–0.515]) and three-spot gourami (mean: 0.432, 95% CI [0.354–0.511]) seemed to have the same reverse perception of this illusory pattern of the other three species but their performance was not statistically significant (Santacà, Lucon-Xiccato & Agrillo, 2020). Consequently no firm conclusion could be drawn regarding their perception of the Delbeouf illusion since the statistic was barely over the threshold for significance (Santacà, Lucon-Xiccato & Agrillo, 2020).

## Discussion

The heterogeneous pattern of results described in the literature on visual illusions in fish (Agrillo et al., 2020) raised the possibility that grouping mechanisms fundamental to perceiving several illusory phenomena may be different among fish. We accordingly investigated whether five different teleost fish tested in identical conditions exhibited different degrees of global-to-local precedence. The overall analysis does not support this hypothesis: fish seem to have a significant global-to-local precedence, with no apparent difference among the species. In addition, their global-to-local preference was not influenced by the shape (circle vs. cross) of the positive stimulus, nor was it related to variations in the spatial arrangements (dense vs. sparse array) of the local elements.

When looking at the species-specific level, one may argue that the species may actually differ in their grouping mechanisms. In fact, zebrafish did not show significant preference, whereas the other four species demonstrated a significant global-to-local precedence. It is worth noting that two previous studies showed that zebrafish have a lower performance in operant conditioning tasks compared to other animal species (Agrillo et al., 2012; Miletto Petrazzini et al., 2019). Hence it is possible that finding the reinforced stimulus in the incongruent condition has a greater effect on zebrafish. Three out of the four species that demonstrated a significant global-to-local precedence seem to have a lower performance in the incongruent condition compared to the performance in the congruent condition. This apparent difference between the performance in the two conditions in angelfish, redtail splitfin and three spot gourami can be due to a lower magnitude of global-to-local precedence compared to Siamese fighting fish. In fact, a global-to-local precedence does not exclude that a species actually processes the local information but only that the global information is processed before the local information. Siamese fighting fish can have a stronger global-to-local precedence compared to the other three species. The possibility also exists that the Siamese fighting fish were unable to see the local cues in the incongruent condition leading to a global bias. To exclude this possibility, this species could be trained to select the local cues of the incongruent condition. If they successfully learn to discriminate the local cues in such an experiment, it would reinforce the conclusion that they can actually process the local information, but they process the global information first. Nonetheless, none of the differences emerged as statistically significant in the analyses. One possible explanation for such an apparent discrepancy could be the limited number of subjects tested per each species or even by the limited number of trials performed by each subject. Testing more subjects for each species or presenting more trials can shed light on how shared the grouping mechanisms are and how much stronger they are between teleost fish.

Our same results were found in the only study that previously investigated the global-to-local precedence in a fish species, the redtail splitfin (Truppa et al., 2010). However, we found a less strong effect of the global-to-local precedence; in fact, in our experiment redtail splitfin demonstrated a preference for the reinforced stimulus at the global level of 56%, whereas in the study of Truppa et al. (2010) they demonstrated a preference of 66%. It is worth noting that our work and the study of Truppa et al. (2010) differed with respect to several methodological factors, such as the types and the duration of the different phases, the number of trials in each phase and the type of reward (food vs. social companions). The same result obtained from two different studies supports the conclusion that performance of this species actually reflects a true global-to-local precedence rather being the results of contextual factors, such as the type of stimuli and procedure, even if these can have an impact on its magnitude.

The fact that fish perceive ‘the forest before the trees’ (Navon, 1977) of course does not exclude that they cannot focus on local details but indicates that, like humans, they tend to prioritize a global analysis of the stimuli, at least in this type of task. In fact, as demonstrated in pigeons, a species can be equally able to process the global and local features of an image, but adopts the global-to-local or the local-to-global precedence as a consequence of the environment or the spatial distribution of the stimuli (e.g., Cavoto & Cook, 2001; Fremouw, Herbranson & Shimp, 1998; Fremouw, Herbranson & Shimp, 2002; Legge, Spetch & Batty, 2009). In different environments, it is possible that some visual information is more salient and important compared to other visual cues. Therefore, the complexity of the habitat might have a crucial role in determining the global or local precedence in a species. It can be inferred that several factors could influence complex processes such as the perceptual grouping mechanisms. For example, in humans, it has been demonstrated that the processing of hierarchical stimuli is related to the elaboration of different spatial-frequency channels (Shulman et al., 1986). In particular, low frequencies code the global structure of an image whereas high frequencies code the local features. The time necessary to elaborate the local features was greater when the global structure was in conflict whereas the time to elaborate the global features was the same irrespective of the local structure (Shulman et al., 1986). This asymmetry suggests a dominant role of low frequencies that make the global elaboration available more quickly compared to the high frequencies that require more time to process the local features. This causes a temporal order from the global processing to the local processing that could be an adaptation of the visual system. In fact, this order makes the perceptual elaboration less susceptible to optical imperfections (Hughes, Nozawa & Kitterle, 1996).

Several factors may favour the sensibility to low spatial frequencies in marine animals. For example, in murky waters, it is difficult to see distant objects and to discriminate precise details. It has been demonstrated that goldfish (*Carassius auratus*) have a higher sensitivity to low spatial frequencies, which has been suggested as possibly due to the adaptation of its wild ancestor to murky waters (Northmore & Dvorak, 1979). When looking at the performance of the single species, four out of five fish species tested in this study demonstrated a global-to-local precedence with no statistical evidence of inter-individual differences. The four species are easily found in murky waters and are phylogenetically closer compared to the remaining species, the zebrafish, which prefers clearer waters (Spence et al., 2006). According to the maximum parsimony criterion, it can be hypothesised that the ancestor of these four species also inhabited murky waters and, consequently, evolved the global-to-local precedence due to a better elaboration of low spatial frequencies. However, it cannot be excluded that the global-to-local precedence is highly variable and that the four species had independently developed it. Nonetheless, data on primates seem to contradict the direct effect of spatial frequency sensitivity in determining the global or local precedence (e.g., Fagot & Barbet, 2006; Fagot & Tomonaga, 1999). Due to the lack of studies on the spatial frequency sensitivity of the fish tested in this study, at present we cannot draw a conclusion regarding such a relationship.

Focusing on the ‘big picture’ might also be adaptive in fish for other reasons. For instance, the capacity to extrapolate the global shape of predators also helps individuals detect them in the presence of cryptic camouflage, a fact that reduces the risks of being captured. Camouflage is indeed a strategy to contrast perceptual grouping mechanisms that lead to the detection of the predator body’s outline by their prey (Troscianko et al., 2009). In addition, we might suppose that perceiving a school of fish as a single Gestalt might lead to benefits in terms of swimming direction, helping individuals to synchronize the speed and direction of their swimming with the rest of the school.

## Conclusions

The mixed results described in the literature on visual illusions in fish raised the possibility that grouping mechanisms that are fundamental to perceiving such illusions may be different among fish. We accordingly studied whether five fish species exhibited a tendency to prioritize a global analysis of a visual stimulus—also known as global-to-local precedence—instead of focusing on local details. The present study suggests that teleost fish may show a robust global-to-local precedence when perceiving hierarchical stimuli. This study also represents a first attempt in exploring the relationship between the grouping mechanisms and the ecological adaptations of teleost fishes and the relationship between the grouping mechanisms and the perception of visual illusions. Due to the large number of factors that could influence the grouping mechanisms more studies are needed, such as the studies that investigate spatial frequency sensitivity that are necessary to understand the effect of this sensitivity on the visual and neural elaboration of hierarchical stimuli. That said, our study suggests that the fact that a species proves to have a different sensitivity to visual illusions does not directly allow the generalization that such species has a different global-to-local mechanism underlying the perceptual representation of hierarchical stimuli.

##  Supplemental Information

10.7717/peerj.9871/supp-1 Supplemental Information 1 Individual data of each species in both congruent and incongruent conditions Individual frequency for the reinforced control stimulus in the congruent condition and for the stimulus chosen in case of a global-to-local precedence in the incongruent condition.

## Additional Information and Declarations

### Data Availability

The following information was supplied regarding data availability: The individual raw data are available as a Supplemental File.
